# Supplementary material for: β-RA Targets Mitochondrial Metabolism and Adipogenesis, Leading to Therapeutic Benefits against CoQ Deficiency and Age-Related Overweight
Source: Biomedicines. 2021 Oct 13;9(10):1457. doi: 10.3390/biomedicines9101457 (PMC8533582; doi:10.3390/biomedicines9101457)
Supplement: Supplementary file 1 [file biomedicines-09-01457-s001.zip › biomedicines-1395487-Supplementary/1-Supplemental Material_Hidalgo-Gutierrez_2021_rev.docx]

**SUPPLEMENTAL MATERIAL**

**Tables**

**Table S1. Markers of hepatic and renal function in the plasma and urine from *Coq9^+/+^* mice and *Coq9^+/+^* mice under 0.33% of β-RA supplementation.** GOT = glutamate-oxaloacetate transaminase; GPT = glutamate-pyruvate transaminase; AP = alkaline phosphatase.

| **PLASMA** | | |
| --- | --- | --- |
|  | ***Coq9*^+/+^** | ***Coq9*^+/+^ + 0.33 % β-RA** |
|  | Mean ± SD | |
| GOT (U/L) | 76.20 ± 21.75 | 66.95 ± 8.48 |
| GPT (U/L) | 41.70 ± 19.63 | 25.20 ± 9.81 ***** |
| AP (U/L) | 24.58 ± 17.86 | 18.77 ± 15.49 |
| Urea (mg/dl) | 143.60 ± 147.93 | 5.97 ± 13.28 ***** |
| Creatinine (mg/dl) | 0.50 ± 0.17 | 0.41 ± 0.086 |
| Albumin (g/dl) | 2.98 ± 0.11 | 2.90 ± 0.089 |
| Bilirubin (mg/dl) | 1.28 ± 0.22 | 1.15 ± 0.26 |
| **URINE** | | |
|  | ***Coq9*^+/+^** | ***Coq9*^+/+^ + 0.33 % β-RA** |
|  | Mean ± SD | |
| Albumin (g/dl) | 0.06 ± 0.026 | 0.03 ± 0.021 |
| Total Protein (g/dl) | 0.80 ± 0.46 | 0.72 ± 0.21 |
| Creatinine (mg/dl) | 31.39 ± 7.71 | 30.40 ± 4.24 |
| Urea (mg/dl) | 78.96 ± 125.65 | 3.72 ± 1.54 |
| Uric Acid (mg/dl) | 1.80 ± 1.70 | 1.72 ± 0.82 |
| Phosphorus (mg/ml) | 66.63 ± 26 | 77.02 ± 18.14 |
| Calcium (mg/dl) | 7.83 ± 4.83 | 6.79 ± 3.07 |
| Magnesium (mg/dl) | 5.47 ± 0.77 | 5.65 ± 1.77 |

**Figures**


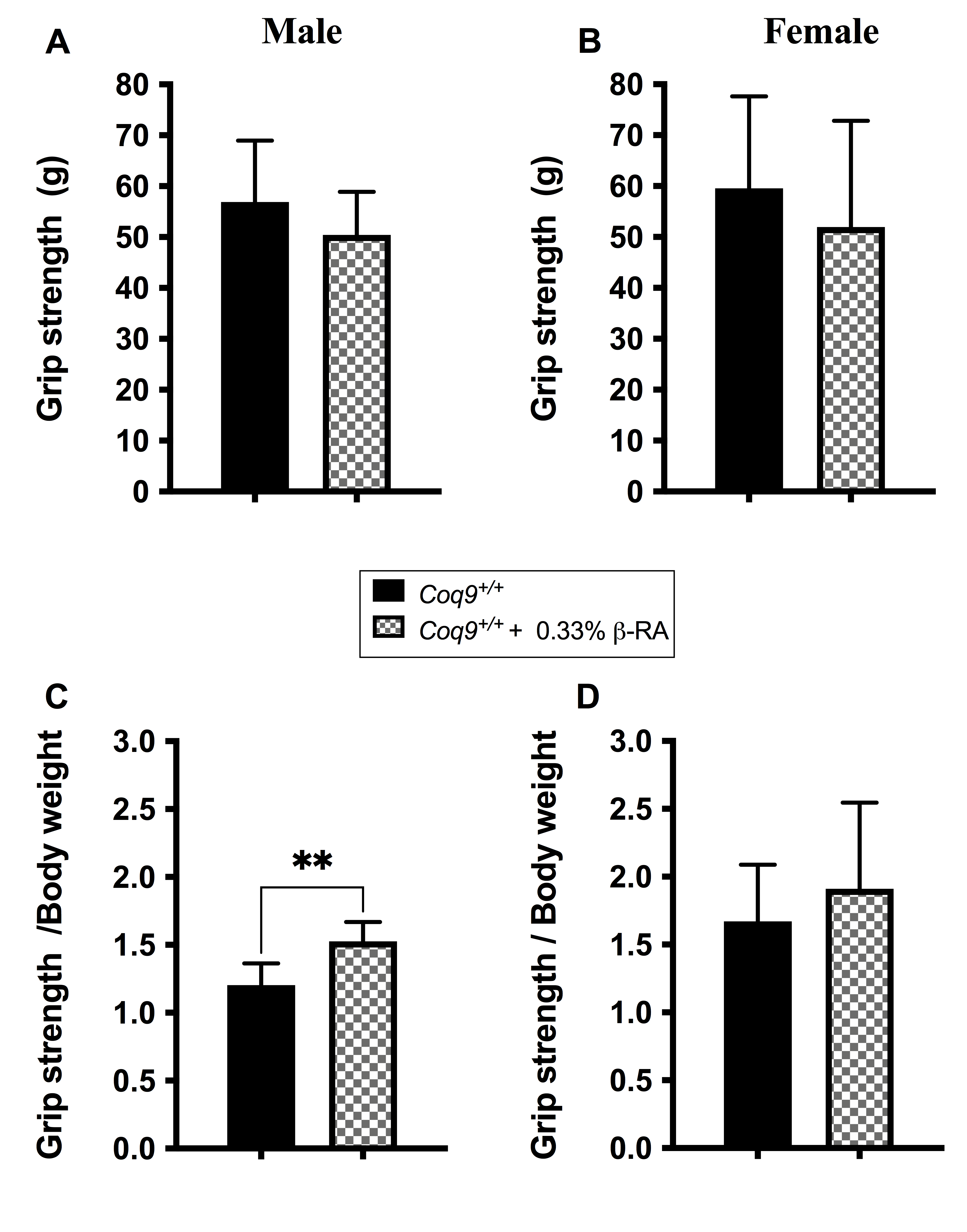


**Figure S1**. **Muscle Strength.**

(**A** to **D)** Grip test in the hind legs of male (A, C) and female (B, D) wild-type mice supplemented with 0.33% β-RA. Data are expressed as grip strength (A, B) and grip strength normalized by the body weight (C, D).

Data are expressed as mean ± SD. **P < 0.01, differences *versus* *Coq9^+/+^* (Mann-Whitney nonparametric test; n = 6–9 for each group).

**Figure S2. Morphological and histological features from *Coq9*^+/+^, *Coq9*^+/+^ mice under 0.33% of β-RA treatment, *Coq9*^R239X^ and *Coq9*^R239X^ mice under 0.33% of β-RA treatment at 3 and 18 Month of age.**

(**A1** and **B1**) Representative images of *Coq9*^+/+^ (A1) and *Coq9*^R239X^ (B1) mice at 3 months of age.

(**C1** to **J1**) H&E and oil-red stains in sections of the liver from *Coq9*^R239X^ (C1 to F1) and *Coq9*^R239X^ mice under 0.33% of β-RA treatment male (G1 to J1) at 3 months of age.

(**K1** to **V1**) H&E and Masson’s trichrome stains in sections of the liver from *Coq9*^+/+^ (K1 to M1) and *Coq9*^+/+^ mice under 0.33% of β-RA treatment male (Q1 to S1) and of kidney from *Coq9*^+/+^ (N1 to P1) and *Coq9*^+/+^ mice under 0.33% of β-RA treatment male (T1 to V1) at 3 months of age.

(**W1** to **H2**) H&E in sections of the spleen from *Coq9*^+/+^ (W1 and X1) and *Coq9*^+/+^ mice under 0.33% of β-RA treatment male (C2 and D2), of heart from *Coq9*^+/+^ (Y1 and Z1) and *Coq9*^+/+^ mice under 0.33% of β-RA treatment male (E2 and F2), and of gut from *Coq9*^+/+^ (A2 and B2) and *Coq9*^+/+^ mice under 0.33% of β-RA treatment male (G2 and H2) at 3 months of age.

(**I2** to **R2**) H&E stains in sections of the diencephalon from *Coq9*^+/+^ (I2 and J2) and *Coq9*^+/+^ mice under 0.33% of β-RA treatment male (N2 and O2); and H&E and Masson’s trichrome stains in sections of the kidney from *Coq9*^+/+^ (K2 to M2) and *Coq9*^+/+^ mice under 0.33% of β-RA treatment male (P2 to R2) at 18 months of age.

**
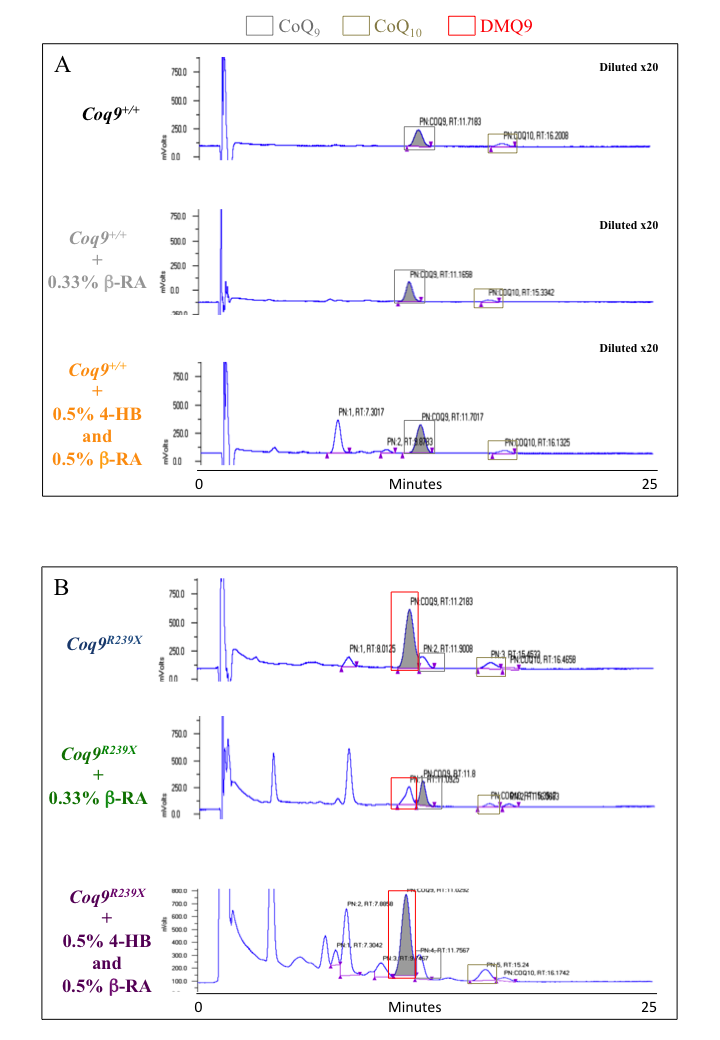
**

**Figure S3. Representative chromatographs showing the peaks of CoQ_9_ and DMQ_9_** **in the kidneys.**

(**A**) Chromatographs for CoQ_9_ in the kidney of a *Coq9^+/+^* mouse*,* *Coq9^+/+^* mouse under 0.33% of β-RA treatment, and *Coq9^+/+^* mouse under 0.5% of 4-HB + 0.5% of β-RA treatment.

(**B**) Chromatographs for CoQ_9_ and DMQ_9_ in the kidney of a *Coq9^R239X^* mouse*,* *Coq9^R239X^* mouse under 0.33% of β-RA treatment, and *Coq9^R239X^* mouse under 0.5% of 4-HB + 0.5% of β-RA treatment.


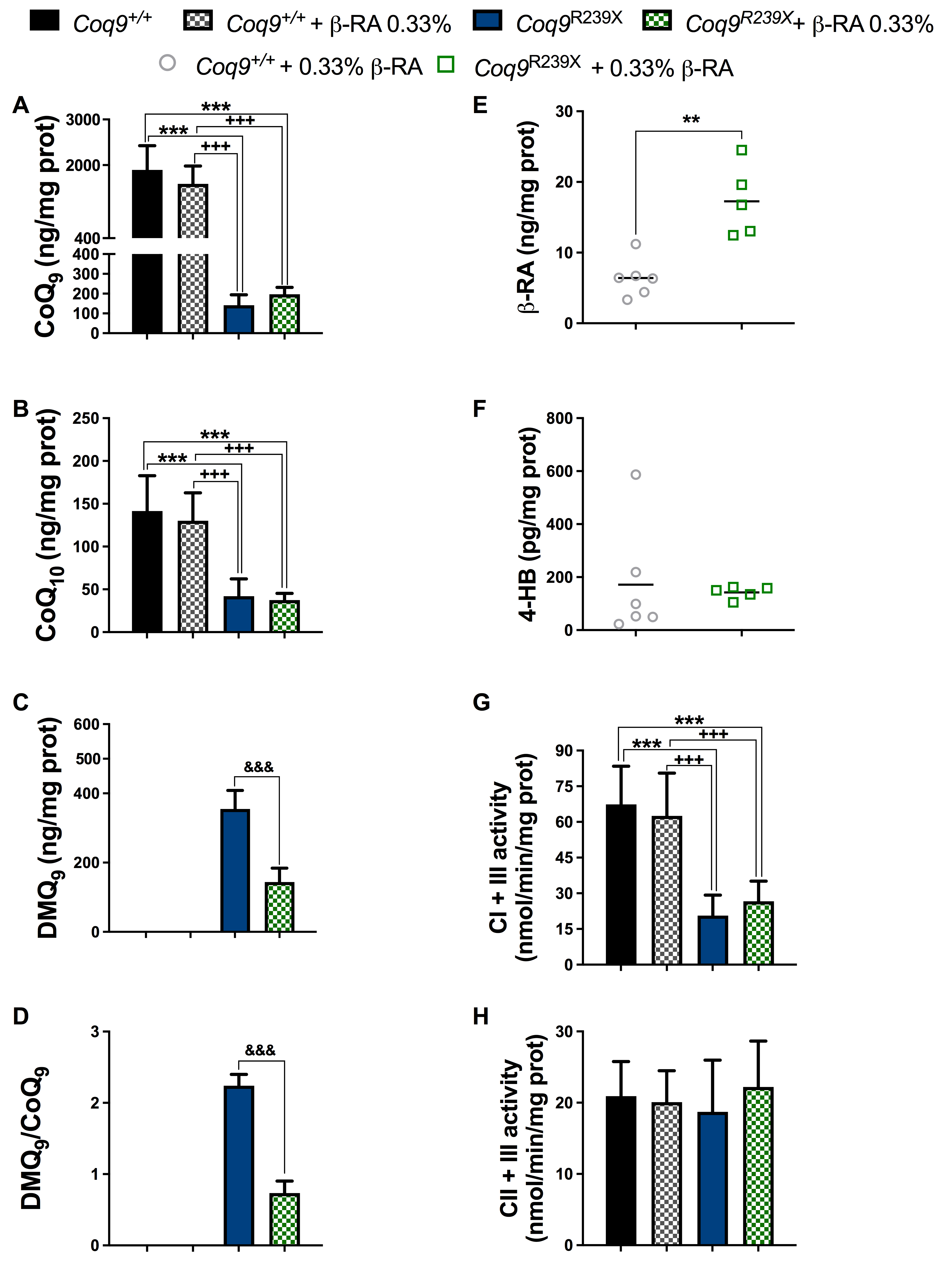


**Figure S4. CoQ metabolism and mitochondrial function in the heart from *Coq9^+/+^* mice*,* *Coq9^+/+^* mice under the supplementation with 0.33% β-RA, *Coq9^R239X^* mice and *Coq9^R239X^* mice under the supplementation with 0.33% β-RA.**

(**A**) Levels of CoQ_9_; (**B**) Levels of CoQ_10_; (**C**) Levels of DMQ_9_; (**D**) DMQ_9_/CoQ_9_ ratio; (**E**) Levels of β-RA; (**F**) Levels of 4-HB; (**G**) Complex I + III (CI + III) activities; (**H**) Complex II+ III (CII + III) activity.

Tissues from mice at 3 months of age. Data are expressed as mean ± SD. **P < 0.01; ***P < 0.001; differences versus *Coq9^+/+^*. ++P < 0.01; +++P < 0.001, differences *versus* *Coq9^+/+^* under 0.33% β-RA treatment (one-way ANOVA with a Tukey’s post hoc test or Mann-Whitney nonparametric test; n = 5–8 for each group). Note that DMQ_9_ is not detected in samples from *Coq9^+/+^* mice.

**Figure S5. CoQ levels in WAT from *Coq9^+/+^* mice *and* *Coq9^+/+^* mice under the supplementation with 0.33% β-RA.**

(**A**) Levels of CoQ_9_; (**B**) Levels of DMQ_9._

Tissues from mice at 3 or 18 months of age (MO). Note that DMQ_9_ is not detected in samples from *Coq9^+/+^* mice.

**
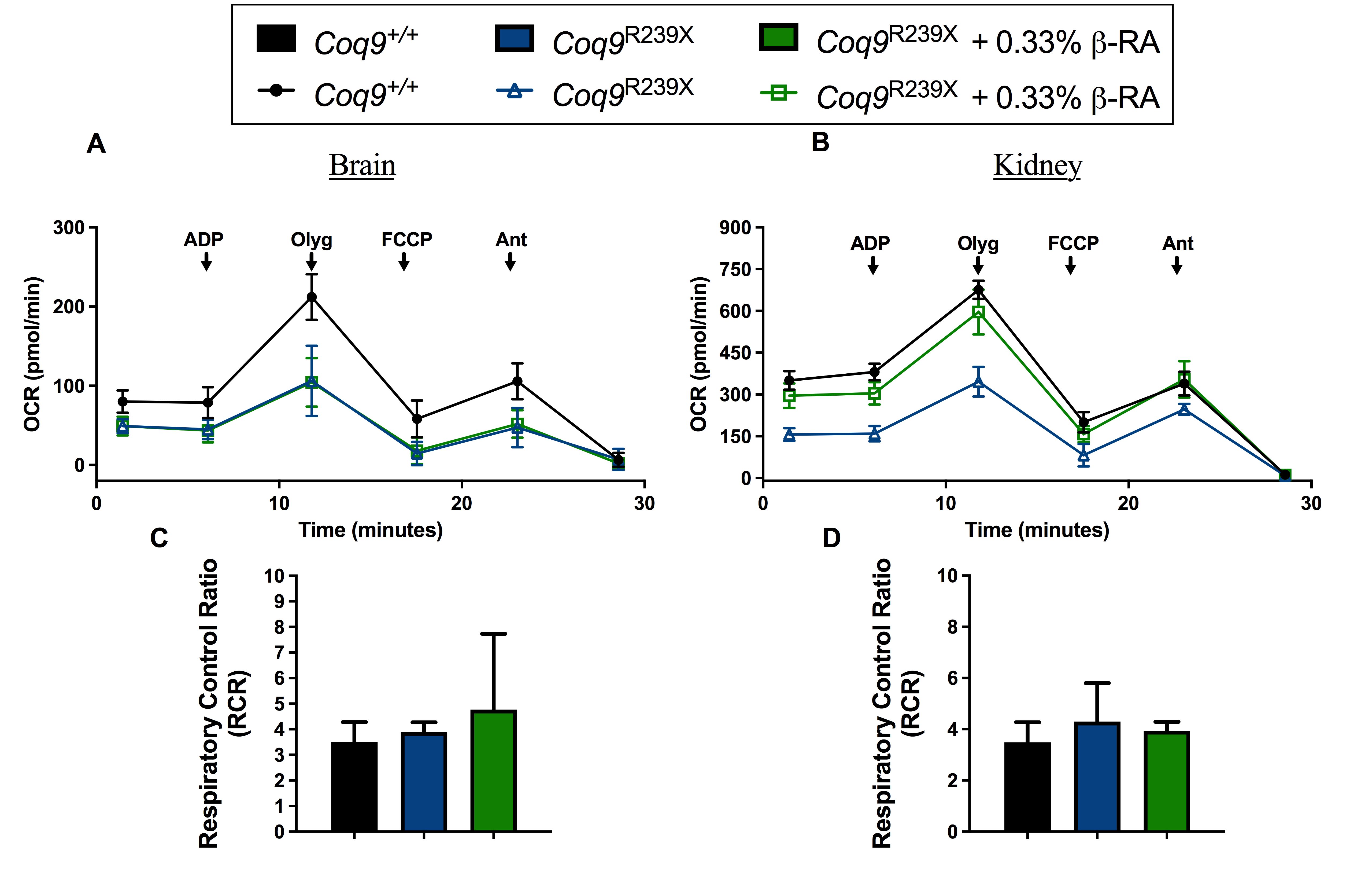
**

**Figure S6. Mitochondrial oxygen consumption rate (represented as State 3o, in the presence of ADP and substrates) in brain (A) and kidneys (B).** ADP = Adenosine diphosphate; Olig = Oligomycin, a mitochondrial ATP synthase inhibitor; FCCP = Carbonyl cyanide-p-trifluoromethoxyphenylhydrazone, an uncoupler of mitochondrial respiration and ATP synthesis; Ant = Antimycin A, a mitochondrial Complex III inhibitor.


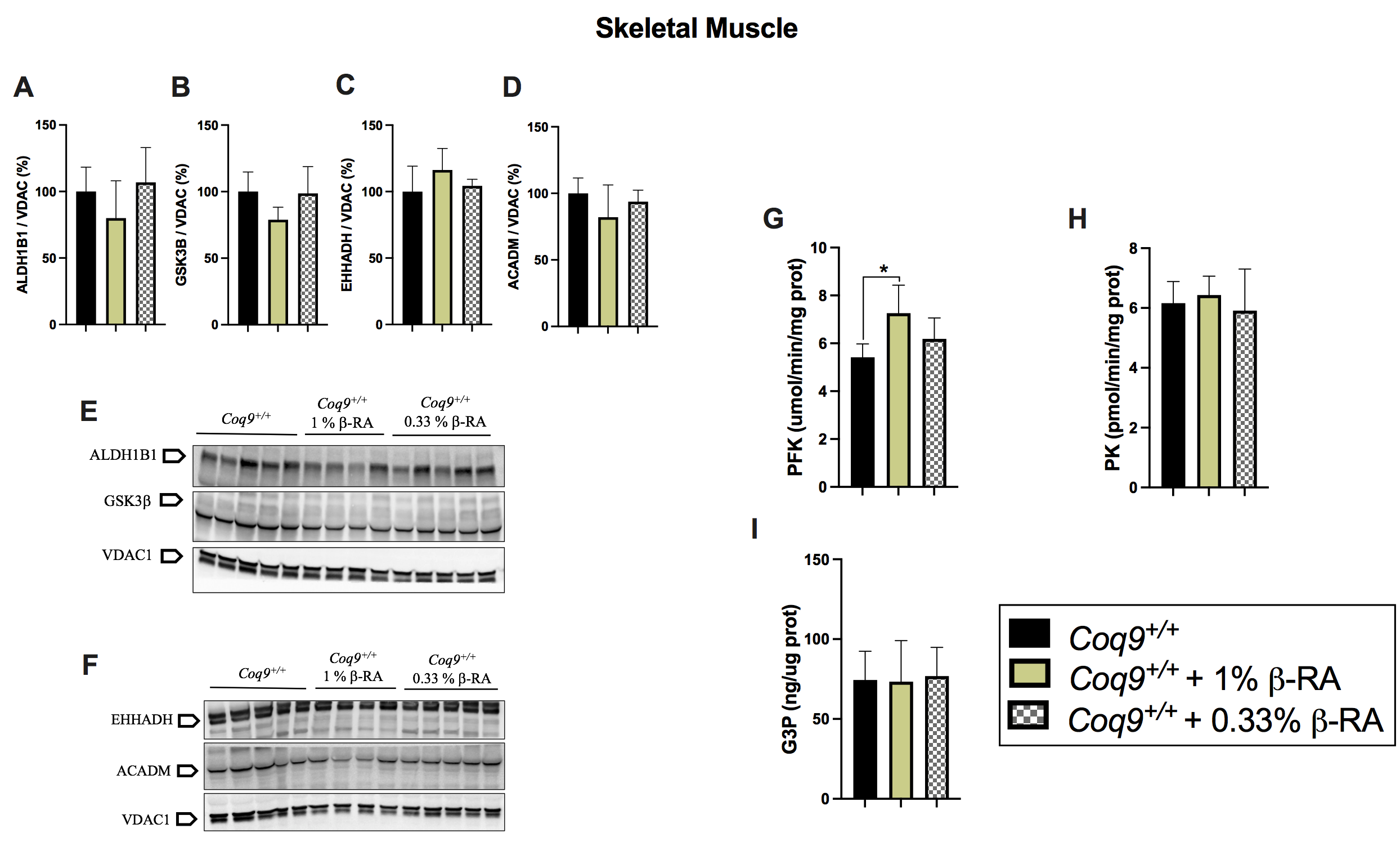


**Figure S7. Metabolic characterization of the skeletal muscle after the treatment with β-RA in *Coq9^+/+^* mice.**

(**A** to **F**) Levels of the proteins ALDH1B1 (A, E), GSK3β (B, E), EHHADH (C, F) and ACADM (D, F) in the skeletal muscle of *Coq9^+/+^* mice treated with β-RA at 1% and 0.33%. VDAC1 was used as loading control. The experiments were performed in tissue homogenate.

(**G** to **I**) Activities of the glycolytic enzymes Phosphofructokinase (PFK) (G) and Pyruvate Kinase (PK) (H) in the and skeletal muscle; levels of Glycerol-3-Phosphate (G3P) (I) in the skeletal muscle (S).

Tissues from mice at 3 months of age. Data are expressed as mean ± SD. *P < 0.05; differences versus *Coq9^+/+^* (one-way ANOVA with a Tukey’s post hoc test; n = 5–7 for each group).


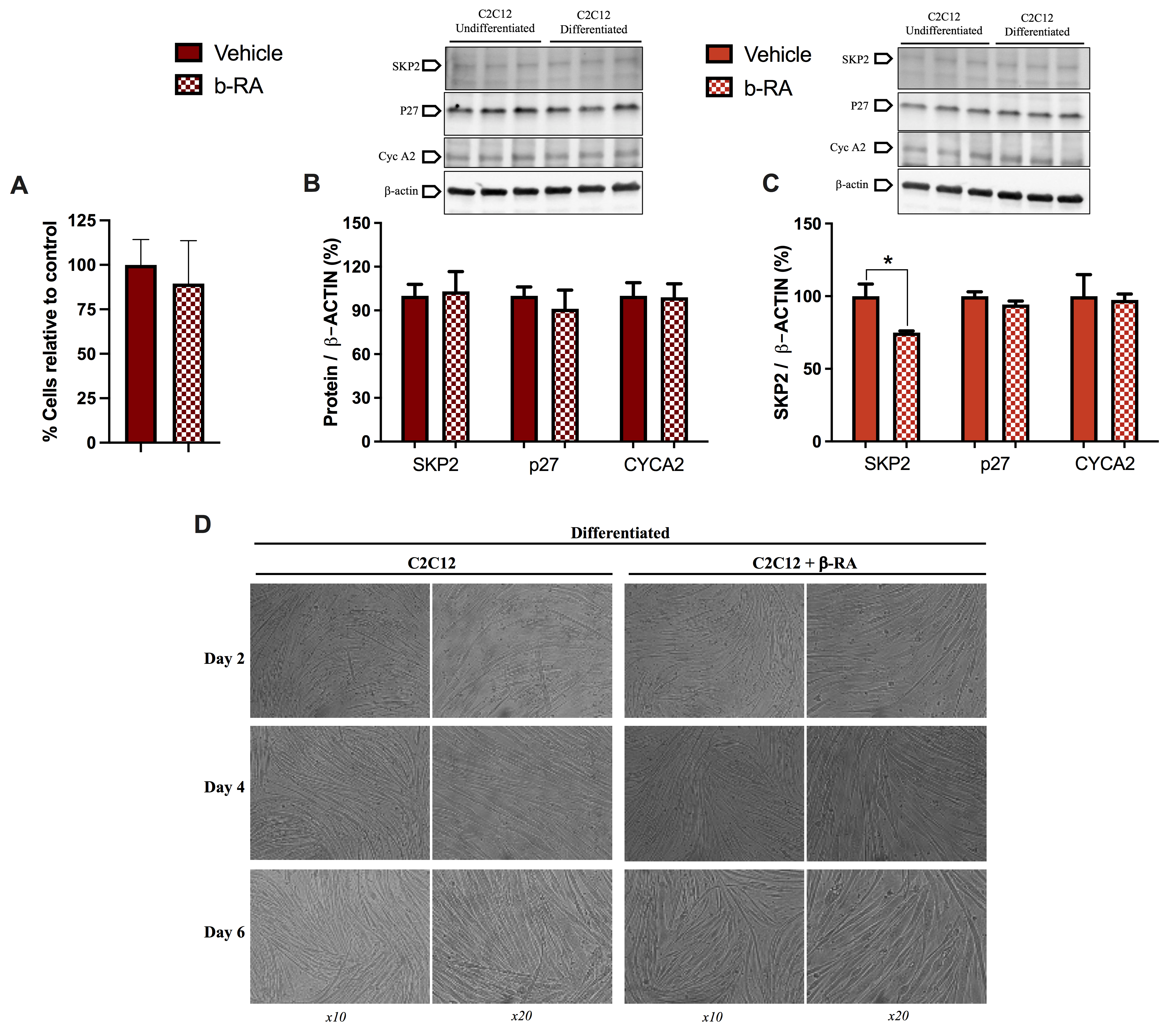


**Figure S8. Effects of β-RA in the proliferation and differentiation of C2C12 myoblasts.**

(**A**) Percentage of C2C12 cells after the treatment with 1mM β-RA, relative to the number of untreated C2C12 cells. Cells cultured in proliferative conditions.

(**B**) Levels of the proteins SKP2, p27 and CYCA2, which are involved in the control of the cell cycle. C2C12 cells were treated for seven days with 1mM β-RA in proliferative conditions.

(**C**) Levels of the proteins SKP2, p27 and CYCA2, which are involved in the control of the cell cycle. C2C12 cells were treated for seven days with 1mM β-RA in differentiative conditions.

(**D**) Images of the C2C12 cells under the microscope in differentiative conditions. C2C12 cells were treated with 1mM β-RA and the images were taken in different days (2, 4 and 6).

Data are expressed as mean ± SD. *P < 0.05, differences *versus* untreated cells (Mann-Whitney nonparametric test; n = 6 for each group).

**
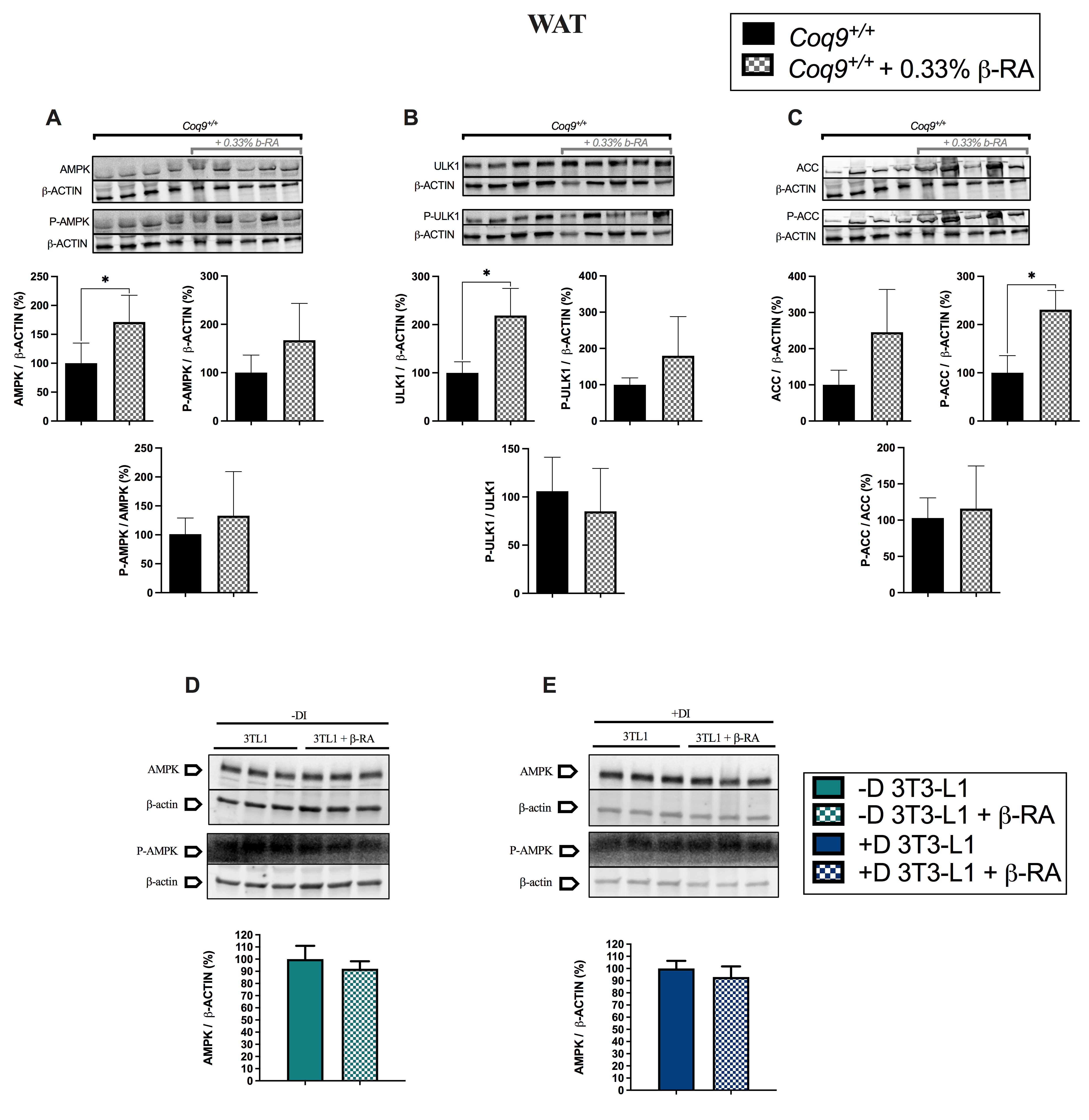
**

**Figure S9. Analysis of the AMPK pathway in white adipose tissues and 3TL1 cells.**

(**A**) Levels of AMPK and p-AMPK, and p-AMPK/AMPK ratio in epididymal WAT from β-RA-treated and untreated wild-type mice.

(**B**) Levels of ULK1 and p-ULK1, and p-ULK1/ULK1 ratio in epididymal WAT from β-RA-treated and untreated wild-type mice.

(**C**) Levels of ACC and p-ACC, and p-ACC/ACC ratio in epididymal WAT from β-RA-treated and untreated wild-type mice.

(**D**) Levels of AMPK and p-AMPK, and p-AMPK/AMPK ratio in 3TL1 cells treated with 1mM β-RA in proliferative conditions for seven days.

(**E**) Levels of AMPK and p-AMPK, and p-AMPK/AMPK ratio in 3TL1 cells treated with 1mM β-RA in differentiative conditions for seven days.

Tissues from mice at 3 months of age (A to C). Data are expressed as mean ± SD. *P < 0.05; *P < 0.05, differences *versus* *Coq9^+/+^* mice (Mann-Whitney nonparametric test; n = 4-6 for each group).

**Legends to Supplementary Videos**

Movie S1. Video that shows the difference between a *Coq9^+/+^* mouse and a *Coq9^R239X^* mouse under 0.33% β-RA supplementation, both males at 20 months of age. Both animals have a healthy appearance, although the treated *Coq9^R239X^* mouse is smaller, as previously reported.

Movie S2. Video that shows the difference between a *Coq9^R239X^* mouse and a *Coq9^R239X^* mouse under 0.33% β-RA treatment, both males at 3 months of age. The untreated *Coq9^R239X^* mouse has developed a paralysis in the legs, although the treated *Coq9^R239X^* mouse has a healthy appearance.

Movie S3. Video that shows a *Coq9^+/+^* mouse and a *Coq9^+/+^* mouse under 0.33% β-RA supplementation, both males at 20 months of age. The appearance of both animals is similar.
